# Social cohesion through football: a quasi-experimental mixed methods design to evaluate a complex health promotion program

**DOI:** 10.1186/1471-2458-10-587

**Published:** 2010-10-05

**Authors:** Sally Nathan, Anne Bunde-Birouste, Clifton Evers, Lynn Kemp, Julie MacKenzie, Robert Henley

**Affiliations:** 1 School of Public Health and Community Medicine, University of New South Wales, Sydney, Australia; 2 School of Public Health and Community Medicine, University of New South Wales, Sydney, Australia; 3 International Communications Department, University of Nottingham, Ningbo, China; 4 Centre for Primary Heath Care and Equity, University of New South Wales, Sydney, Australia; 5 School of Public Health and Community Medicine, University of New South Wales, Sydney, Australia; 6 San Carlos Apache Tribe Wellness Center, San Carlos, USA

## Abstract

Social isolation and disengagement fragments local communities. Evidence indicates that refugee families are highly vulnerable to social isolation in their countries of resettlement. Research to identify approaches to best address this is needed. Football United is a program that aims to foster social inclusion and cohesion in areas with high refugee settlement in New South Wales, Australia, through skills and leadership development, mentoring, and the creation of links with local community and corporate leaders and organisations. The Social Cohesion through Football study's broad goal is to examine the implementation of a complex health promotion program, and to analyse the processes involved in program implementation. The study will consider program impact on individual health and wellbeing, social inclusion and cohesion, as well as analyse how the program by necessity interacts and adapts to context during implementation, a concept we refer to as plasticity. The proposed study will be the first prospective cohort impact study to our knowledge to assess the impact of a comprehensive integrated program using football as a vehicle for fostering social inclusion and cohesion in communities with high refugee settlement.

**Methods/design:**

A quasi-experimental cohort study design with treatment partitioning involving four study sites. The study employs a 'dose response' model, comparing those with no involvement in the Football United program with those with lower or higher levels of participation. A range of qualitative and quantitative measures will be used in the study. Study participants' emotional well being, resilience, ethnic identity and other group orientation, feelings of social inclusion and belonging will be measured using a survey instrument complemented by relevant data drawn from in-depth interviews, self reporting measures and participant observation. The views of key informants from the program and the wider community will also be solicited.

**Discussion:**

The complexity of the Football United program poses challenges for measurement, and requires the study design to be responsive to the dynamic nature of the program and context. Assessment of change is needed at multiple levels, drawing on mixed methods and multidisciplinary approaches in implementation and evaluation. Attention to these challenges has underpinned the design and methods in the Social Cohesion through Football study, which will use a unique and innovative combination of measures that have not been applied together previously in social inclusion/cohesion and sport and social inclusion/cohesion program research.

## Background

### Introduction

Australia accepts more than 13,000 refugee and humanitarian immigrants annually[1]. Worldwide there were 42 million displaced people, including 15.2 million refugees by the end of 2008 [2]. There is evidence that refugee families are highly vulnerable to social isolation in their countries of resettlement [3]. The difficulties of refugee settlement are well documented, including the need to learn new languages, negotiating differing cultural and societal values, emotional trauma, loss or separation from family and torture or life-threatening events preceding arrival [3-6]. The Australian government and public are in the midst of a debate about how to foster settlement that enables humanitarian refugees to overcome barriers that currently hinder their participation in Australia's social fabric [7].

Youth account for a large overall percentage of the refugee population in New South Wales (NSW). For all refugee and humanitarian entrants arriving in NSW (the most populous state in Australia) in the last five years, 50% were under 20 years of age, with 69% of entrants under 30 years of age - these figures are even higher in the two program sites for the current study [5]. Settlement issues, together with the 'normal' challenges youth encounter in their stage of personal development - changing family and peer relationships, education pressures, and increasing independence - make young refugees particularly vulnerable to health and social difficulties, affecting their capacity to trust and their relationships with family, teachers, peers and the broader community [3-6].

In recent there has been an increase in programs addressing the social dimension of refugee settlement, but there is a paucity of robust impact assessment both in Australia and internationally on these programs [3]. Well researched programs are needed to address the complex challenges to socially cohesive refugee settlement [3,5,6].

### A role for sport?

There is evidence of the positive impact of sport on individual participants' physical, mental and social health [8-12]. At the community level, sport has been advocated as a mechanism to promote a socially cohesive society [10,12], encourage strong community bonds, reduces crime rates, and offer access to positive mentors [8,11,13-15]. These positive impacts are particularly important for communities with high numbers of young people from disadvantaged backgrounds [8,16-19]. Researchers point to the potential for sport to build relationships and social cohesion across religious, ethnic and economic lines, but there is little hard evidence to support this assertion [9,15,20,21].

Moreover, while the positive impact of participation in sport on individuals and communities is widely promoted, the phenomena that can bond teams and groups around their sport can also exclude individuals and groups and further divide communities [20-22]. Tonts cautions that sport experiences can have adverse effects if not designed and implemented carefully to foster positive relationships and provide bridging mechanisms [21]. Perkins et al's [23] review of youth programs in the US notes that ethnic minority youth, particularly those living in economically distressed communities, do not participate equally in organised sport. This means they can become alienated from broader national and community networks and support that enable access to social and economic resources. A recent paper by Hutchins pointedly critiques the idea of sport as a mechanism for building social cohesion [24]. Hutchins sets out three reasons why a cautious eye is needed when assessing sport and its potential for promoting social cohesion. These reasons are that 1) sport is historically divisive and can create an 'us' and 'them' mentality strengthening intra-group bonds and oppositional identity; 2) social cohesion on the field may not translate to 'enduring' social cohesion in the broader community; 3) the internal logic and structure of sport is competitive with rules and regulations promoting winners and losers which can act against community-building and cross-cultural understanding. Care clearly needs to be taken in *how *programs are developed and implemented to ensure that they promote team work, cross-cultural understanding and that they are socially inclusive rather than exclusive, including actively working to provide bridges to mainstream community organisations and structures and vice-versa [8-10,25].

Football programs for social and human development are on the increase both in Australia and internationally [8-10,17,26]. Anecdotal and early research indicates the potential of such programs [9,10,25,27], but the mechanisms by which they can influence social inclusion and social cohesion need comprehensive and rigorous investigation [8-10,25,28]. Scholars in the field highlight the need to evaluate sport-based programs that directly address social inclusion to assess their effects and inform future initiatives [8-10,25]. Coalter's review [8] of the evidence of the impact of sport on individual and community level outcomes such as health, crime, employment and regeneration found the evidence was largely anecdotal and there was a lack of robust studies to inform the design of sport programs which aimed to foster social inclusion. Recent reviews provide further support for this criticism [8,9].

### Football United

Football United (FUn) is a complex health promotion intervention that uses a football (soccer) development program as a mechanism for promoting individual health and well being, and fostering social inclusion and cohesion in areas of Sydney, Australia with high refugee settlement [8,9,29]. Complex interventions or programs are those which include several interconnecting components [30]. The FUn program is underpinned by an ecological framework recognizing that health and social behaviour are influenced at multiple levels by societal and structural variables, and social processes [31,32]. Programs to promote individual health, well being and social inclusion, and foster social cohesion need to intervene at all of these levels and include capacity building elements such as training, mentoring, leadership and partnership development in order to achieve maximum impact. To achieve this, the Football United program operates in partnership with migrant and refugee support organisations, football organisations, schools, corporate and community groups.

The Program elements are integrated into three key focus areas:

1. Football activities: These include regular Saturday and after school programs, gala days and school holiday camps. All activities are designed to foster maximum mentorship between coaches and players, between older and younger players, and between volunteers and participants.

2. Capacity building: Youth and family members from the communities have opportunities to participate in courses and apply their learning in the following areas: coaching and refereeing, mentoring and life-skills, leadership, first aid, project management and volunteering.

3. Fostering involvement with local football clubs and linkages between the program and partner agencies - government, community and corporate.

The different activities have been designed to provide participants with skill sets that are transferable to other contexts such as fund raising, club management and development, volunteer management and support, and to promote linkages between newly arrived youth and families and other community members, families, community, corporate and sports organisations.

The program is innovative because of its complexity in addressing many levels of social inclusion and cohesion thereby addressing the criticisms of past sport for social development programs as lacking such complexity [19,21-23,25]. Football United directly involves newly arrived refugees from up to 20 different countries at any given time, settled community members, and partners from the broader community in designing and running the program [10,17,21]. The choice of football as the vehicle in this program is purposeful and particularly significant - it is relatively inexpensive, it enjoys worldwide enthusiasm, it is designed as a non-violent, non-contact sport, is played by both genders and is the sport of choice among many in the refugee communities where the program is implemented [26].

## Methods/design

### Aims and hypotheses

The Social Cohesion through Football study's broad goal is to investigate the implementation of FUn over a three-year period to analyse processes and impacts on individual health and wellbeing, social inclusion and cohesion. For the purpose of this study, the term social cohesion is defined as "the ongoing process of developing a community of shared values, shared challenges and equal opportunity ... based on trust, hope and reciprocity" [33]. Our understanding of the term incorporates elements of social inclusion which "is characterised by a society's widely shared social experience and active participation, by a broad and equality of opportunities and life chances for individuals and by the achievement of a basic level of well-being for all citizens" [34]. We do note that it is impossible to share some values as cultural, religious, ethnic, gender, generational, sexual, geographical and bodily specificity maintain difference, however we take the expression "shared values" to mean not necessarily "the same" but rather negotiation and mutual exploration of different and similar values.

The study has four aims and two hypotheses.

#### Aims

1. To determine the impact of Football United on participants' personal development, emotional health, resilience, social inclusion, peer relationships and other 'life skills'.

2. To determine the impact of Football United on social cohesion in the school and broader community.

3. To investigate issues arising from implementation of the program in order to inform future program implementation and replication of the program in other contexts, including other Australian and international sites.

4. To establish guidelines for best practice sports-based social inclusion and cohesion programs.

#### Hypotheses

1. Participants in the Football United program will have significantly better emotional health, peer relationships and feelings of social inclusion and those who do not participate at all or who only participate minimally in the program.

2. The Football United program will contribute to social cohesion in communities with high refugee settlement.

### Study design

The central element of the study will be a quasi-experimental cohort study design with treatment partitioning which will be used to accommodate the particular challenges of working with a study population that is changing (new immigrants arrive regularly and move locations) as well as ethical dilemmas related to excluding possible participants from the program. It employs what could be termed a 'dose response' model, comparing those with no involvement in the Football United program (at a single point in time enabling them to then participate in the program following measurement) with those with lower or higher levels of participation. Treatment partitioning involves separating the program group into low and high levels of participation in FUn activities. The study design reflects the reality that settlement is an ongoing process, and that different young people will access the program at different time. In addition, the program has already been running for more than two years in the study sites. The combination of these factors means that a true baseline measurement is precluded. A cohort study design with treatment partitioning is the best approach to enable causal inference and protect internal validity [35].

The program group will be students in two Intensive English Centres (IECs) in Sydney, Australia who have participated in the FUn program in the study year. IECs are part of the school system in the state, and prepare newly arrived, secondary aged students for study in an Australian high school by providing intensive English tuition [36]. At the end of data collection and prior to data analysis program participants will be allocated to the low or high level of participation group based on their actual participation levels during the study period. The comparison group will be recent arrivals to Australia who are attending an IEC in another location where FUn is not currently operating. They will need to have been in Australia for at least 3 months and have not participated in any FUn program activity. The comparison group will be recruited to match the program group as closely as possible, including their cultural background. All school-aged participants will be recruited to the study directly through their respective IEC. The views of key informants in the school and wider community, as well as volunteers and paid workers in the program will also be explored.

### Measures

#### Individual impact measures

A range of qualitative and quantitative measures will be undertaken with all consenting IEC participants in the study year. Study participants' emotional well being, resilience, ethnic identity and other group orientation, feelings of social inclusion and belonging will be measured using a survey instrument complemented by relevant data drawn from in-depth interviews, self reporting measures and participant observation.

##### Survey instrument

In designing the survey instrument, a number of existing instruments were considered for each key construct of interest based on a review of the literature around these constructs and their measurement. The composite survey instrument was agreed upon after a number of discussions among the research team where survey instruments were compared across a range of criteria, including match to construct of interest, previous use (including in other languages), availability of data on psychometric properties, such as reliability and validity, length, availability of national comparative data and cost to use. Instrument face validity was also considered by the researchers and key partners who work with the study population. For example, some instruments included colloquial Anglo-Saxon expressions which would not translate well into other languages and cultures. Table 1 shows the instruments chosen and the rationale for inclusion in the composite survey instrument.

**Table 1:** Instruments and items chosen for survey

Instrument/items	Construct measured	Rationale for choosing
Strengths and difficulties questionnaire - SDQ[74]	Emotional symptoms, conduct problems, hyperactivity/inattention, peer relationship problems pro-social behaviour and intervention impact.	• Match to constructs of interest high. • Widely used in non-clinical populations. • Published psychometric data available [75]. • Translated and used in over 50 languages. • Australian comparative data available [76]. • Language simple and clear compared to other related scales. • Each of the sub-scales includes 5 items allowing separate scores to be calculated. • Use is free.
Connor-Davidson Resilience Scale (CD- RISC)[77]	Resilience	• A short 2 item version CD-RISC2 available helping to reduce composite instrument length [78]. • Published psychometric data available including on shorter version [79]. • Wording appropriate for a non-clinical adolescent population. • Other alternative (The Resilience Scale) had a number of items similar to the SDQ and is 14 items long [80]. • Usage fee US$150
Multigroup Ethnic Identity Measure[81-83] and Other Group Orientation Scale[84]	Ethnic identity	• Two scales are available that measure ethnic group identity and other group orientation. • Published psychometric data available [85]. • No translations available. • Used in the Good Starts Study - Victoria, Australia[37,38]. • Use is free
Selected items from the Health and Participation Survey from the Building Healthy Communities: Health Development and Social Capital Project[86]	Feelings of social inclusion	• 3 items (12,13,15) chosen that examine connections in their neighbourhood with family and friends. • Designed for adult population so questions chosen were most appropriate for young people. • No national data, but smaller geographic areas available. • Psychometric data not available, as instrument does not measure a personality or mental health construct. • Use is free.
Selected items from NSW Child Health Survey[87]	Feelings of social inclusion	• Designed for parents/carers of children - 2 items chosen (246, 250) which were modified for young people. • Has been translated and used in other languages. • NSW parent data available. • Psychometric data not available, as instrument does not measure a personality or mental health construct. • Use is free.

A range of demographic data are to be collected in the survey, such as age, country of birth, language spoken, date of arrival, country prior to arrival, level of English using the same questions, where possible, as the recent Good Starts Study in Victoria, Australia [37,38]. These data will be used to describe the samples and to control for confounders in analysis. Nine practice questions have been designed and included at the beginning of the survey to demonstrate the different types of questions and response options in the survey. Practice questions are then repeated at the beginning of the set of questions which use the same response options. The layout and design of the instrument includes pictures of young people and is intended to be appealing to those who have little experience with survey forms and low literacy levels. The survey will be administered with bilingual support as needed and the need and type of assistance recorded on the survey forms.

##### Friendship pair interviews and self reporting

Friendship Pairs (FP), where a selected participant chooses a friend to be interviewed with them, will provide a comfortable and supportive environment for the young people and encourage them to speak freely without undue peer pressure often apparent in focus groups [39]. A first FP interview will be undertaken with a sub-sample of participants soon after survey administration and will be focussed on their experiences settling in Australia (in both program and comparison schools) and their experiences of the FUn program (program schools only). At the first interview, following a 'self-reporting' approach, the FUn participants will be given scrapbooks in which they will be asked to write, paste images or draw about what they like and dislike about the FUn program and living in Australia. They may also be given a disposable camera to record their experiences. They will be asked to return the scrapbook and disposable camera prior to the second interview. The physical act of this 'self-reporting' has been shown to enable quieter or less assertive young people to have their feelings and observations represented in the study and produce their own frame of meaning [40]. This material will be used to prompt discussion in a second interview.

A second interview will be undertaken with those from the program group two to three weeks following the first interview. In the second interview researchers will explore the narratives the participants construct about the scrapbooks and photographs, as well as consider the themes and perspectives the participants do not include [41]. For participants who are not as comfortable articulating their experiences verbally, they are likely to be able to represent their experiences visually with far greater depth and richness ensuring their views are captured in the study [42-44]. The use of photographs has well established credentials in facilitating engagement in research among these types of participants [45-48]. The field researchers will also spend time with the young people at both comparison and program schools during the study period to build trust and rapport while also observing school settings, program implementation and participants' experiences.

Better outcomes for the program group could be explained by their participation in the research itself rather than the program, a phenomenon known as The Hawthorne Effect [49,50]. To address this, only a sub-sample of the program group will actively participate in the friendship pair interviews and the self-reporting exercise, allowing this group to be compared to the group who participate in the program, but are only involved directly in the research via the survey measures at a single point in time.

##### Participant observation

Participant observation of Football United activities will include recording numbers of youth, parents, volunteers and community members engaged in FUn activities at the two schools and will document the different roles of the various participants and stakeholders and the dynamics of engagement. Interaction between youth, coaches, volunteers, community leaders and other significant stakeholders will be documented. This data will complement and add further depth to the data collected from other methods about impact and allow the research team to observe the experience of the program in real-time [51,52]. Direct observation will also allow the collection of data about contextual factors in the school settings that influence the program.

#### Community context measures

The context and surrounding environment of each of the schools will be assessed primarily through Key Informant (KI) interviews at two points during the study period. Data from participant observation and FP interviews will also provide insights into contextual factors. KIs will include key people in the school, such as the principal and head teacher, as well as other staff who will be identified as key informants using snowball sampling [51]. People in key agencies, such as Migrant Resource Centres and youth organisations who interact with, and provide services to the school will also be identified and interviewed. In addition, the operation of FUn and views about the program will be examined among KIs at the program schools including FUn coaches, staff and students who have long term involvement with the program. Lastly, community leaders or elders in relevant community/ethnic groups for all the four schools will be interviewed about their community issues and concerns. KIs' knowledge of, and views about FUn will also be explored in program schools. The broader societal context will also be measured using media coverage in state, local and niche language media outlets. These media will be examined to identify and assess coverage of refugee and settlement issues in general and coverage and discussion of FUn activities focussing in on both number of occurrences as well as content.

#### Process measures

Process measures will be undertaken to document the issues arising from implementation and for future replication of the program. Measures will examine process issues at two levels: 1) local implementation of the program activities, and 2) program management at advisory group level. Data sources will include: participant observation, friendship pair interviews, self report data, KI interviews, meeting transcripts and minutes.

### Ethics and informed consent

Ethics approval has been received from the UNSW Human Research Ethics Committee and the NSW Department of Education and Training Student Engagement and Program Evaluation Bureau [53]. The consent process is being undertaken with partner organisation input. The study population is vulnerable and some may have experienced persecution in their homelands, sometimes in the form of governmental and bureaucratic abuse. The process of gaining consent from young people and their parents will entail careful negotiation and management to ensure any intrusion into their lives is minimised whilst enabling meaningful and informed consent.

### Sampling

The survey sample for the program group (across the two schools) is estimated to be approximately 100-150 youth representing all youth who participate in the program and consent to inclusion in the study. With treatment partitioning in the program group, the sample will have power of greater than .80 to detect small to moderate effect sizes. Approximately 100 young people will be recruited as a comparison group for the survey. An additional benefit of the study design is that there will be no need to follow a large number of the program or comparison group for further survey measures meaning there will be no loss to follow-up.

The recruitment of a subsample of the FUn program group to participate in in-depth interviews and self-reporting aspects of the study will employ purposeful sampling to ensure both diversity and richness of the data collected [51]. Participants who are sampled will be asked to nominate a friend in the program to be interviewed with them in the friendship pair. The number of participants required for the qualitative aspect of the study will be determined by that needed to reach saturation of the data [50]. The number of participants needed to reach data saturation varies depending on the variety of experiences associated with the phenomenon, however saturation is expected with the inclusion of around 20 participants (10 paired interviews per site) [50,54]. A sub-sample of the comparison group will also be recruited to participate in a friendship pair interview after the survey administration using purposeful sampling.

Initial KI interviewees will be purposefully selected and snowball sampling then utilised to identify further key informants within and beyond the school context [51]. Participant observation is planned to occur every week at each of the two program schools during school playing activities and at other selected program activities as they occur, such as training programs and Gala days.

### Data Analysis

#### Quantitative analysis

Descriptive analysis of the demographic characteristics of the program and comparison groups and univariate analysis of measures, such as emotional well being, ethnic identity, and resilience will be undertaken. Although matching will be applied during the recruitment processes for the comparison group, it is recognised that the three groups (comparison, low participation and high participation) may differ on the distribution of factors impacting on outcomes, for example, length of time in Australia or age. Propensity score analysis will be used to balance any differences in groups [55,56].

To compare the impact measures across groups, Analysis of Variance (ANOVA) will be used for scale impact measures and Chi-square test will be used for proportional impact measures. In addition, factorial ANOVA will be used to determine interactive effects of demographic variables on measured impacts.

#### Qualitative analysis

Framework analysis will be used for qualitative data [57,58]. Interviews will be recorded on digital media and transcribed along with participant observation notes, and any diagrams/sketches which will be scanned. Analysis will begin concurrently with data collection. The two field researchers will familiarise themselves with the transcripts, notes and images, immersing themselves in cycles of reflection as the key ideas, themes and concepts "crystallise" [59]. Identified themes and concepts will be coded using NVIVO8 [60].

The field researchers will first code a purposeful sample of data selecting information with rich and diverse content which will assist in the construction of theory [51,61], before coming together with the other researchers who will facilitate the comparing, contrasting and negotiation of a content/concept related index. This triangulation of index systems between the researchers will lead to a thematic framework that will be used to code the remaining data. Any discrepancy between indexing the remaining data and the index will be discussed among the researchers. Once all the data is coded by the index system the research team will discuss the resulting thematic framework and examine the nature of the themes and concepts, mapping out relationships and exploring relevant theory to provide explanations for the findings. The survey data will also be compared to the interview data for the sub-sample involved in all measures to triangulate and examine similarities and differences in the findings from the different methods used. Key events and stories in the media coverage for the study period which are focussed on refugee settlement and the FUn program will be analysed with particular attention to how stories and debates are framed and whose views and experiences are represented [62].

The research team has decided not to impose any particular theories in the qualitative data collection process or in the initial analysis, preferring to take an inductive approach [51]. The research findings will not be framed up-front by specific theories, beyond a socio-ecological model and constructivist perspective [31,32,51] allowing the views and meanings from the participants' perspectives to be captured.

## Discussion

### The significance of this study

From our review of the literature, the proposed study will be the first prospective cohort impact study in Australia and internationally assessing the impact of a comprehensive integrated program using football as a vehicle for fostering social inclusion and cohesion in communities with high refugee settlement [9,10,15,17,21,25]. It will both assess impact and examine and document the processes and experiences which underpin the program. The research findings will advance the international knowledge base about how football, and sport programs more generally, can contribute, or not, to fostering social inclusion and social cohesion as well as promote individual health and well being [9,10,15,20,25]. We hope that the study will directly inform the development, implementation and impact assessment of football, and sport programs more generally, in Australia and other countries and contexts with high refugee settlement numbers, or high numbers of disaffected youth.

The study will provide new knowledge on methodology and measures for assessing the impact of sport in vulnerable and culturally diverse populations. The study proposes a unique and innovative combination of measures which have not been applied together previously in social inclusion/cohesion and sport and social inclusion/cohesion program research [9,15].

### Implementing a complex program across different sites

Football United is a complex health promotion program which has been developed with the community over four years and its "active components" or key intervention functions [30,63,64] have been described earlier in this paper. However, the way the program is delivered and how each of the elements is operationalised varies at different sites [63-65]. Strategies to achieve the program goals also change in response to the changing context and community needs over time [66]. We emphasise here that this is what we refer to as the 'plasticity' of the intervention and this characteristic is viewed by the research team to be an element that is advantageous to the proposed success of complex interventions. Plasticity, as we use the term, refers to the capacity of programs and peoples to alter their action and experiences in response to changes in their environment and context. This property can be studied at the micro- and macro-level of program implementation.

The goal in implementation is not to standardise the program across multiple sites, but allow it to respond and interact with context while ensuring the "active components" are present at each site. This is a similar approach to that taken by Hawe et al [67], who argue that the *function *that a component plays in an intervention should be standardised, rather than the form this component. This approach rejects the need for rigid standardisation of programs across various sites: "Indeed, the more complex an intervention becomes, the more it is necessary to have rigorous theory about the process and principles of the change process being tested, but to be flexible about the form that this takes in each site" [67] (p267). This argument is akin to that made by those advocating a theoretical rather than procedural fidelity approach to evaluating program implementation [68] and the many calls for greater exposition of the underlying theories of complex interventions, assumptions about how they work and expected impacts [65,66]. This responsive approach, however, does provide some challenges in undertaking evaluation.

### Evaluating complex health promotion programs

Complexity poses challenges for measurement and evaluation because program impact will often not be straightforward or linear [69]. Too heavy an emphasis on measuring 'outcomes' will also not capture adequately the effects of a complex health promotion program nor provide an understanding of the effects of context. Paterson suggests conceptualizing change as resulting from the interaction between the intervention, process and context over time [65]. Measuring this change requires close attention to the context of the intervention - complex health promotion programs occur in a context, a context which is often integral to the success of the intervention and cannot be separated from its effects [63,66]. Assessment of change is also needed at multiple levels [70] drawing on mixed methods and multidisciplinary approaches in implementation and evaluation of a program [71,72]. Attention to these challenges has underpinned the design and methods utilised in the Social Cohesion through Football study. The desired plasticity of the intervention will be mirrored by a flexible and emergent study design [51], in particular in participant recruitment and methods application.

Firstly, the overall evaluation design for this study must navigate a range of practical and ethical issues which are unavoidable in the 'real world' of intervention research whilst also providing the strongest design possible to allow causal inference. A cohort study design with treatment partitioning and propensity scoring provides the best approach to enable causal inference and protect internal validity whilst taking account of the specific community and program context [35]. Context specific information that is particularly significant for this study includes the fact that the program has been well-established in the study sites for two or more years and that the study population is fluid with new arrivals and departures every school term.

Secondly, the study design must concurrently capture program impacts at a range of levels and document contextual factors at the school and community level that may influence program shape and implementation, and mediate impacts. Comparisons across the four schools will be critical to building understanding of the role of context as part of the intervention and to enable the context and plasticity of the intervention to be considered in interpreting impact data. The program operates in an ever-changing environment where predicting, let alone controlling variables that may affect the outcomes being studied, is impossible [66], and also not ethical. For example, other support and settlement programs run in both program and comparison schools. It would not be ethical to isolate the young people from these programs to narrow causal influence.

Thirdly, the study design is not only focused on measuring outcomes, but will also document and examine the implementation of the program to inform the implementation of similar programs in other settings. In short, we need to keep an eye on what is happening, and how it is happening [66] - the "essence of the intervention" [64] (p1561).

In conceptualising possible impacts, it is important to note that while we will build understanding of the processes of, and interactions between the program components, we also aim to measure outcomes of the whole program and not its constituent parts. This is best practice when evaluating an ecological program impacting on a range of areas at many levels [64,70]. Impacts are hypothesised to occur at 1) the individual level, that is among those who participate in Football United as players, coaches and in other voluntary and paid capacities, 2) the community level, that is, within the school environment, and possibly even 3) beyond to the local geographic community. We need to actively examine whether outcomes accrue only for individuals or if the program has some benefits beyond to the school and broader community as we have hypothesised. Others have noted that community level change (for example in the school environment) cannot be equated with the sum of individual changes [66,73] and we plan to look beyond aggregated individual change.

**Figure 1:**
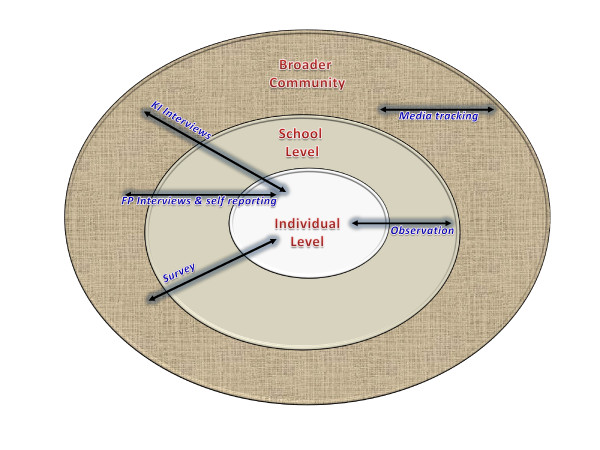
**Levels of impact and measurement tools**.

As a research team we have chosen to utilise multiple methods to try to capture change at the individual, school and community level as well as build our understanding of how the program works and interacts within each specific context. Figure 1 shows how the different measures will provide data across these levels of possible impact. The survey, friendship-pair interviews and self reporting mechanisms will be focussed on the impacts on the wellbeing of young people in the program, and will also tell us about their feelings of social inclusion, trust and cross-cultural relationships in the school and broader community where they live. The impacts of Football United specifically will also be explored with the program school students. The in-depth friendship-pair interview data will also help us to interpret and in a sense 'validate' or question the quantitative findings providing rigour through methods triangulation [30,51,68,71].

Key informant as well as friendship-pair interviews will provide the research team with important insights into the school context and how Football United operates and affects participants and those who deliver the intervention in the two program schools. Interviewees will include the school participants, coaches, teachers, program workers and community agency staff involved, as well as community leaders. Others have noted the importance of such a dialogue with the participants and program workers, and the research team will question and further explore what intervention 'success' means and the role of the participants as active partners in implementation [63,73].

Careful recording of activities implemented and 'exposure' to the intervention has also been recommended in the field of evaluating complex interventions [63]. Process measures which are part of overarching program management for FUn will be used to track activities and participant exposure which will be corroborated by key informant and friendship-pair interviews. Participant observation at the Football United schools will also provide further data to understand what works in the field and what doesn't as part of the intervention, including the role of the participants [63]. Others have lamented the lack of documentation of the actual process of complex interventions [66]. Our study directly addresses this lack of documentation through the use of multiple methods focussed on the implementation of the intervention at all stages.

Key informants will also tell us what is happening in the broader community that may impact on the young people and whether the program effects are felt beyond the school walls. Lastly, the media coverage of Football United will provide a measure of reach beyond the schools involved. The intensity of coverage of the issues of asylum seekers and refugee settlement in the media will also provide an understanding of the broader social and political setting within which the program is operating.

## Conclusion

This study of a complex health promotion intervention delivered at multiple sites addresses many of the documented failures of past studies to grapple with the "plasticity" of such interventions, which varies at each site. By plasticity we mean the ability, or not, to adapt and alter to accommodate the ever-changing conditions of possibility in which interventions are implemented and play out - economic, social, biological, cultural, political, spatial, and the like. Multiple lenses and methods are being deployed to cut through the inherent messiness of this movement, complexity and alteration, and provide rigour through triangulation. The implementation of the program and the role of participants in its success will be analysed. Multiple levels and measures of process and impact, including the individual, school and broader community are included in the study design, which provides a strong model to enable causal inference, but also the ability to analyse how the essence of the intervention responds in a real world context.

## Competing interests

The authors declare that they have no competing interests.

## Authors' contributions

SN, ABB, CE, LK, and RH all contributed to the study design. SN drafted the manuscript and all authors contributed to editing and refining the arguments presented in the final manuscript.

## Authors' Information

SN is a social scientist who uses qualitative and quantitative methods in her research within a participatory framework where research participants are actively involved in the process of research and constructing meaning.

ABB is a social scientist who specialises in design, implementation and evaluation of innovative community development and health promotion scientific and technical programmes at national and international levels. Her particular areas of intervention are youth, disadvantaged and vulnerable communities.

CE researches gender, media, sport and cultural studies at the University of Nottingham, Ningbo, China. He's an active member of numerous community groups and editor of 'Altitude: An e-journal of emerging humanities work'.

LK is a health equity researcher who focuses on mixed method trials of interventions to improve health and wellbeing outcomes for vulnerable communities and populations.

RH is a psychologist who uses quantitative methods in his research, and is interested in the research of resilience processes in youth from different cultures.

JM is a cultural researcher whose research interests include the interaction of gender, rights, and cultural difference.

## Pre-publication history

The pre-publication history for this paper can be accessed here:

http://www.biomedcentral.com/1471-2458/10/587/prepub
